# Supplementary material for: The influenza virus PB2 protein evades antiviral innate immunity by inhibiting JAK1/STAT signalling
Source: Nat Commun. 2022 Oct 21;13:6288. doi: 10.1038/s41467-022-33909-2 (PMC9586965; doi:10.1038/s41467-022-33909-2)
Supplement: Supplementary file 1 — Supplementary Information [file 41467_2022_33909_MOESM1_ESM.pdf]

The Influenza Virus PB2 Protein Evades Antiviral Innate Immunity by Inhibiting JAK1/STAT Signalling

Hui *et al.*

Supplementary Information

Inventory of Supporting Information

1. Supplementary Figures and figure legends

Supplementary Fig. 1 Flow cytometry gating strategy for Fig. 1g.

Supplementary Fig. 2 Densitometry analysis of immunoblots shown in Fig. 2.

Supplementary Fig. 3 IAV PB2 protein promotes degradation of JAK1.

Supplementary Fig. 4 IAV PB2 protein increases the K48-linked ubiquitination of JAK1.

Supplementary Fig. 5 JAK1 Kinase domains interaction with IAV PB2 protein.

Supplementary Fig. 6 Intracellular distribution of IAV PB2 protein in infected A549 cells.

Supplementary Fig. 7 AIV PB2 protein mediates ubiquitination degradation of chicken JAK1 (chJAK1).

Supplementary Fig. 8 Quantitative analysis of mRNA, cRNA, and vRNA levels in AIVs-infected cells.

Supplementary Fig. 9 PB2 protein from different subtypes of AIV promotes degradation of JAK1.

Supplementary Fig. 10 Schematic representation of IAV PB2 protein antagonizes antiviral immunity.

2. Supplementary Table 1 The qPCR primers sequence in this study.

1. Supplementary Figures and figure legends

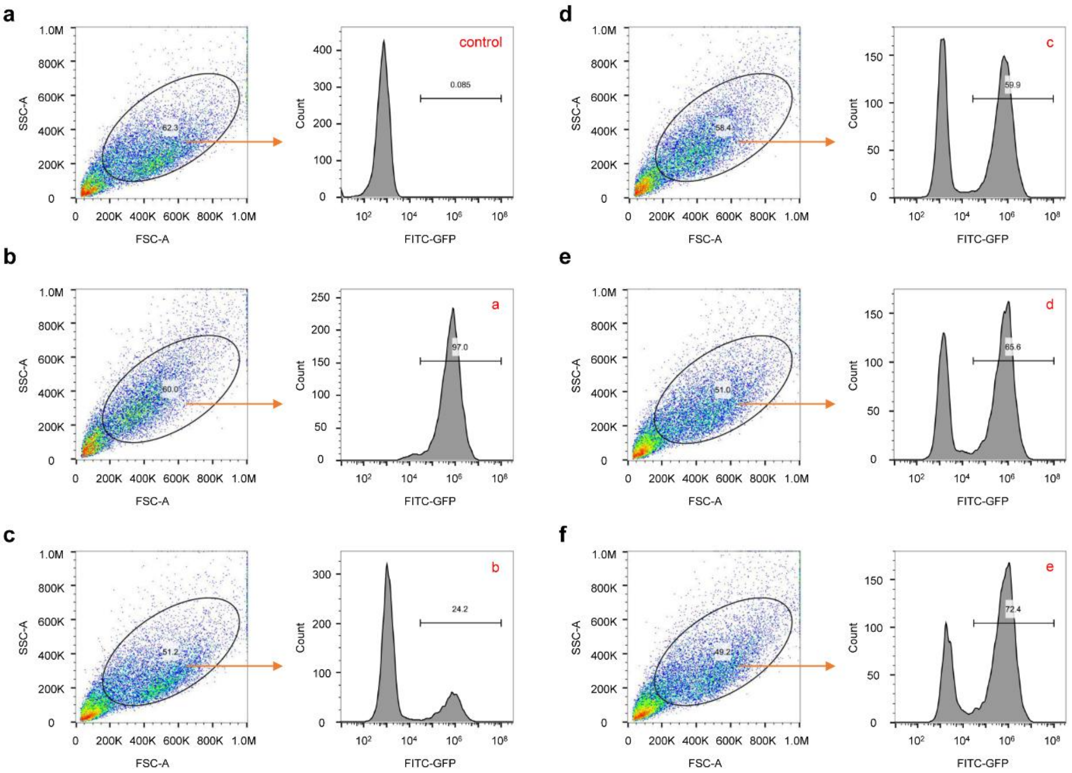

Supplementary Fig. 1 | Flow cytometry gating strategy for Fig. 1g. All Cells were gated based on size and granularity using FSC-A vs SSC-A.

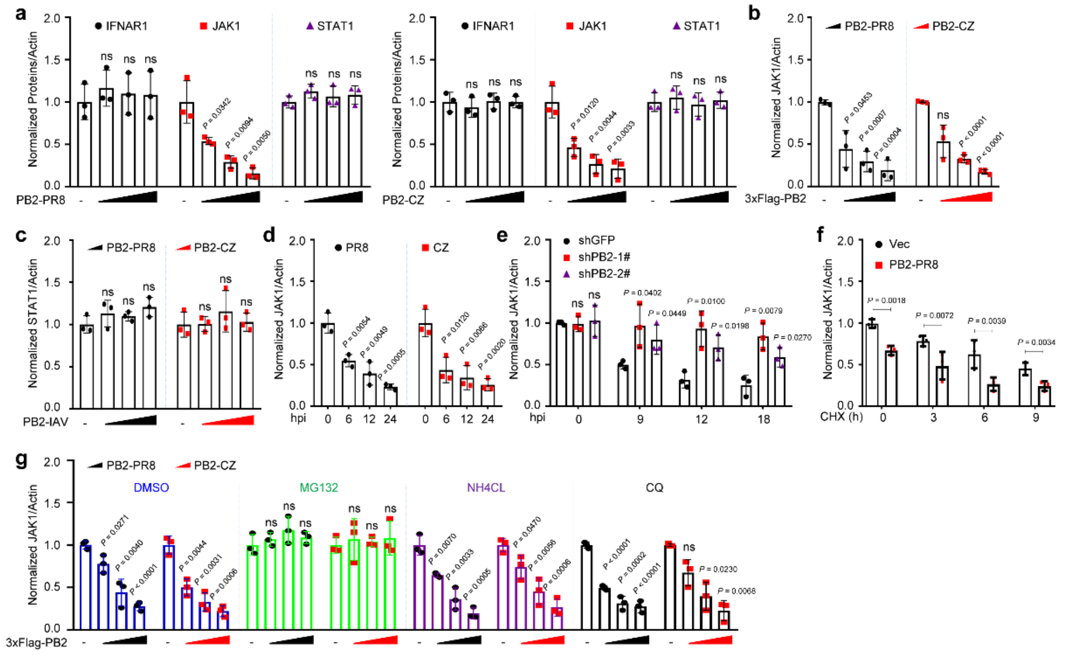

Supplementary Fig. 2 | Densitometry analysis of immunoblots shown in Fig. 2. The intensities of the bands on the immunoblots from three independent experiments were quantified and normalized with actin. Data are presented as means ± SD and statistical significance was determined by unpaired two-tailed Student's *t*-test. ^{ns} P >0.05.

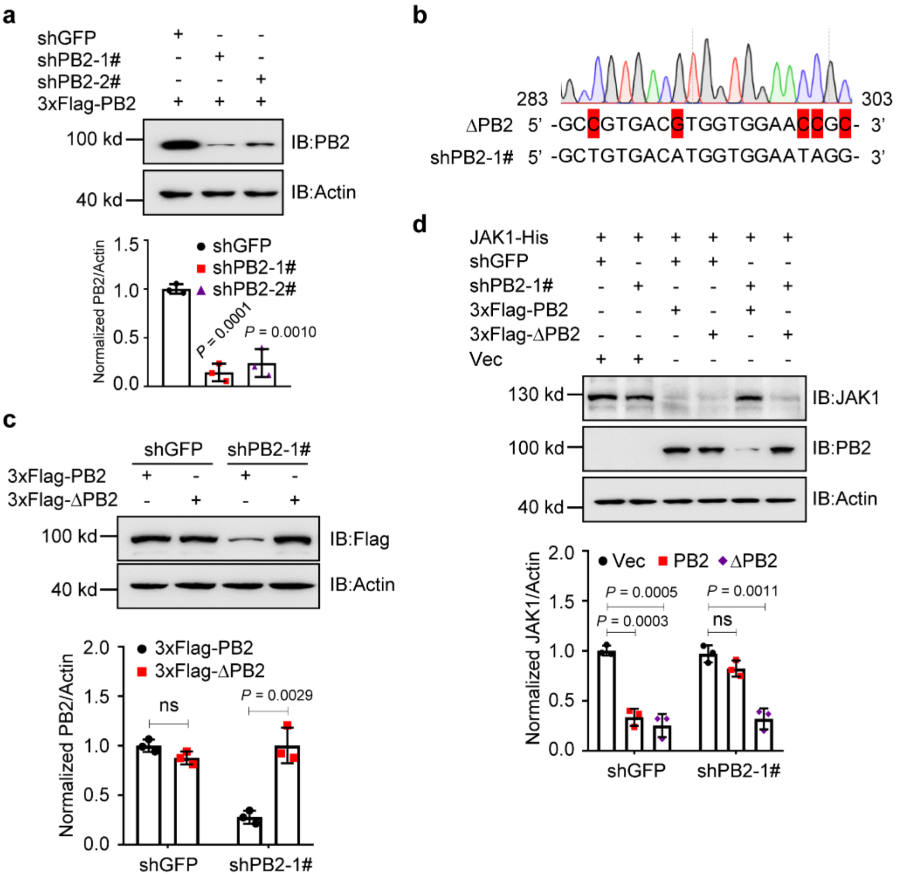

Supplementary Fig. 3 | IAV PB2 protein promotes degradation of JAK1. (a) Immunoblots of HEK293T cells transfected with control shRNA (shGFP) or shRNA targeting PB2 (shPB2) and 3xFlag-PB2-PR8 (upper). The intensities of the bands on the immunoblots from three independent experiments were quantified and normalized with actin (lower). **(b)** The shRNA off-target PB2 mutant (ΔPB2) was constructed by a 5-nucleotide nonsense mutation in the target sequence of the shPB2 plasmid. **(c)** Immunoblots of stable PB2 knockdown HEK293T cells transfected with PB2 or ΔPB2 plasmids (upper). The intensities of the bands on the immunoblots from three independent experiments were quantified and normalized with actin (lower). **(d)** Immunoblots of stable PB2 knockdown HEK293T cells transfected with JAK1-His and PB2 plasmids (upper). The intensities of the bands on the immunoblots from three independent experiments were quantified and normalized with actin (lower). Data are presented as means ± SD and statistical significance was determined by unpaired two-tailed Student's *t*-test. ^{ns} P >0.05.

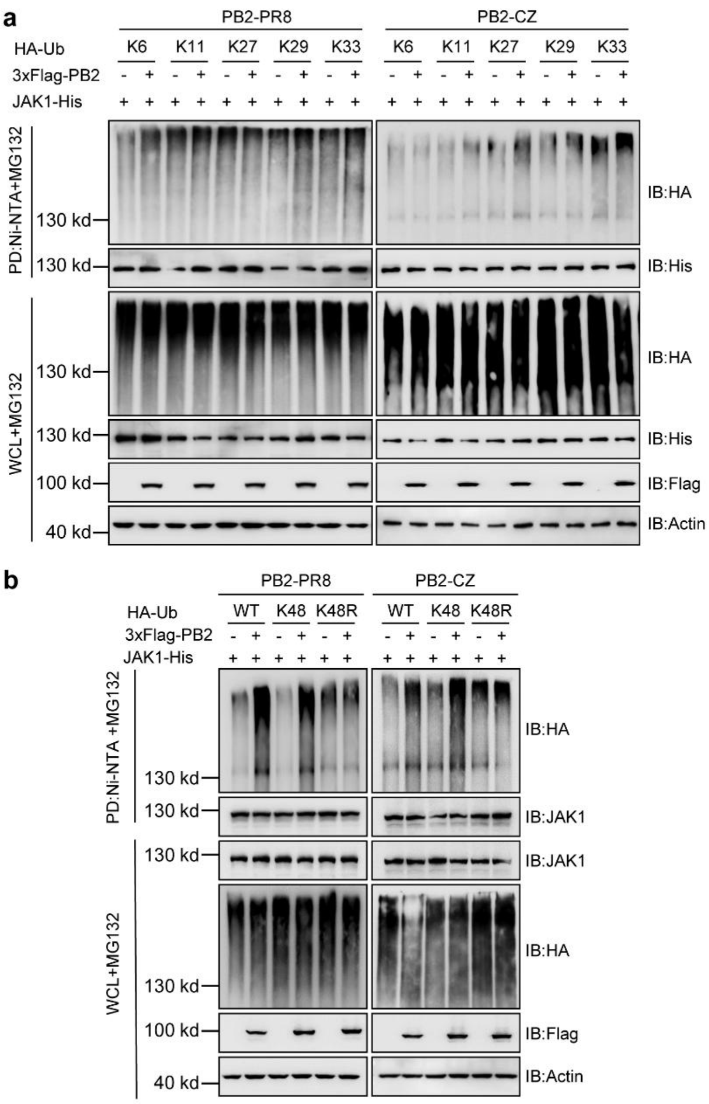

Supplementary Fig. 4 | IAV PB2 protein increases the K48-linked ubiquitination of JAK1. (a) Ni-NTA pull-down analysis of the ubiquitination of JAK1 in HEK293T cells transfected with JAK1, HA-Ub or its mutants, and PB2 plasmids and treated with MG132. **(b)** Ni-NTA pull-down analysis of the ubiquitination of JAK1 in HEK293T cells transfected with JAK1, HA-Ub-K48 or HA-Ub-K48R, and PB2 plasmids and treated with MG132. WCL, whole-cell lysates. Data are one representative of three independent experiments.

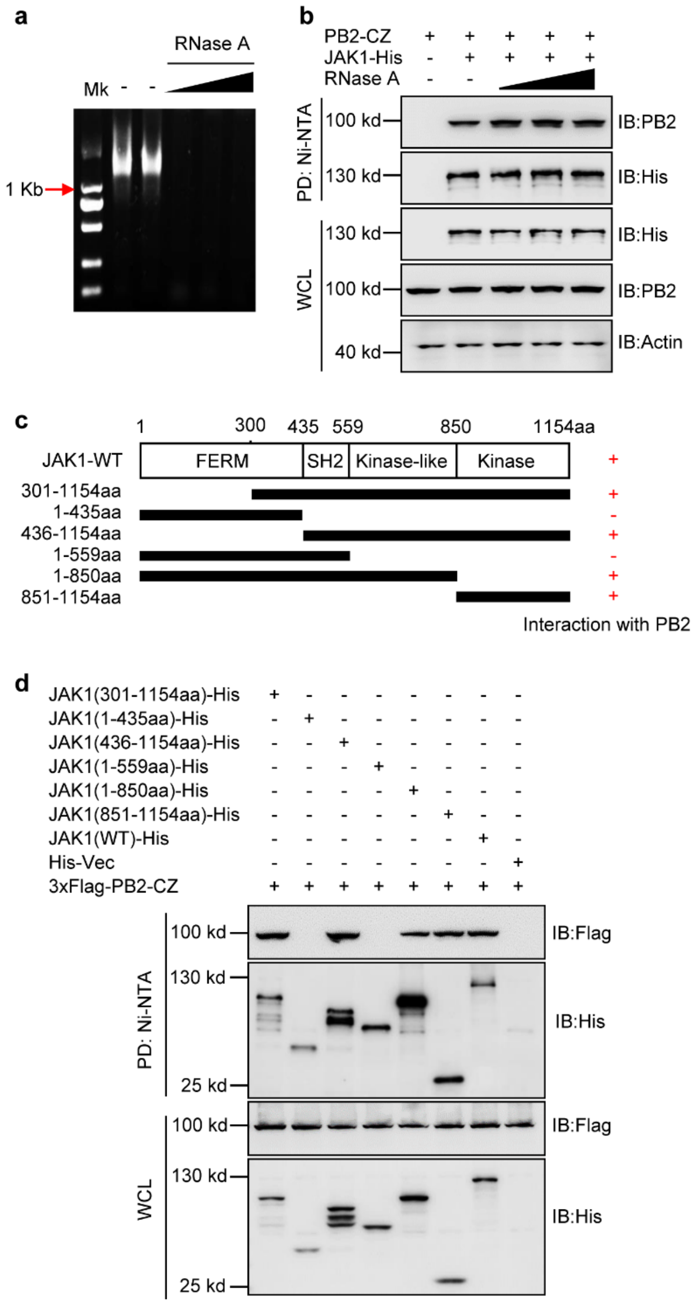

Supplementary Fig. 5 | JAK1 Kinase domains interaction with IAV PB2 protein. (a) Agarose electrophoresis analysis of HEK293T cells treated with RNase A. MK, marker. **(b)** Ni-NTA pull-down analysis of the interaction of PB2 and JAK1 in HEK293T cells transfected with PB2-CZ and JAK1 plasmids and treated with RNase A. **(c)** Schematic representation of the deletion mutants of JAK1**. (d)** Ni-NTA pull-down analysis of the interaction of PB2-CZ and JAK1 or its deletion mutants in HEK293T cells. WCL, whole-cell lysates. Data are one representative of three independent experiments.

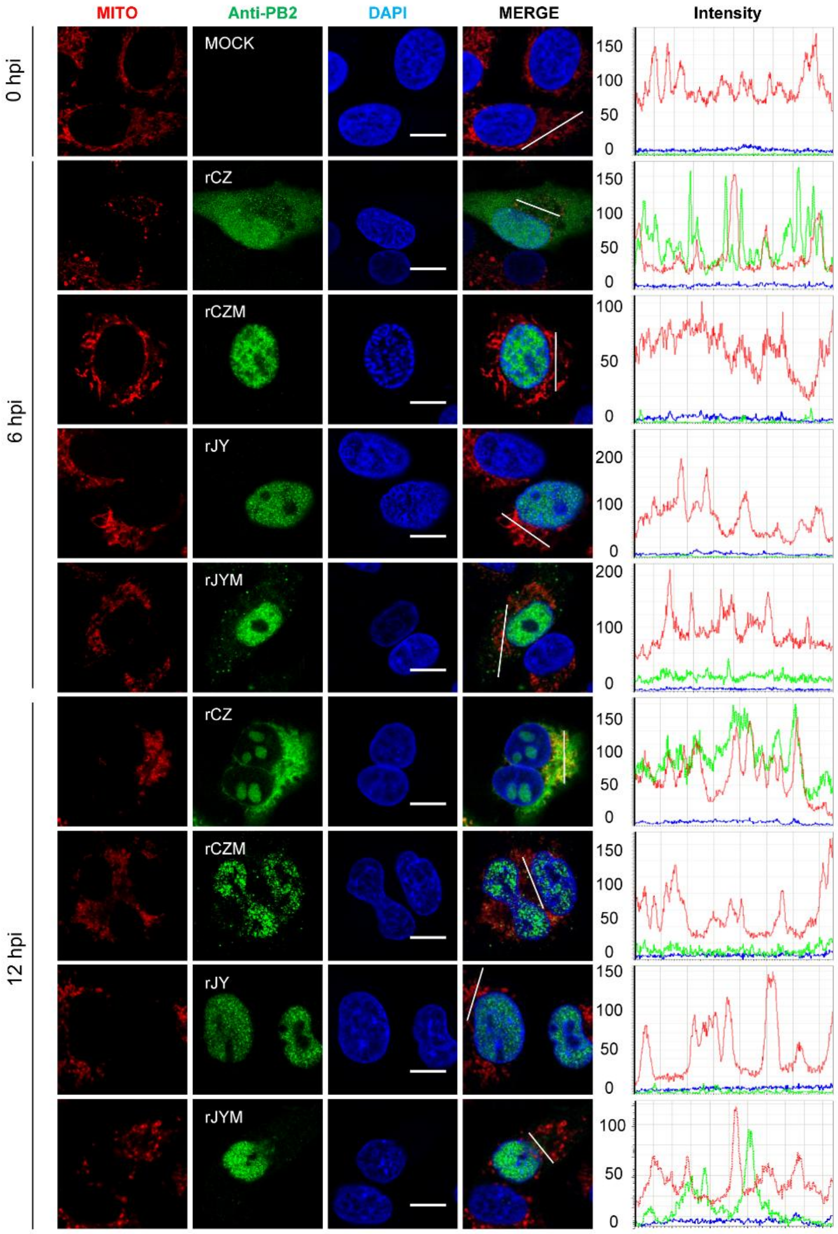

Supplementary Fig. 6 | Intracellular distribution of IAV PB2 protein in infected A549 cells. A549 cells were infected with H5N8 viruses at an MOI of 1. Cells were stained with MitoTracker Red (MITO) to label the mitochondria and PB2 proteins with a rabbit anti-PB2 pAb and Alexa Fluor™ 488 Goat anti-rabbit IgG (H+L). The nuclei were stained with DAPI. Scale bar: 10 μm. hpi, h post-infection. Intensities of fluorescence at indicated locations were scanned by LAS X Software. Data are one representative of three independent experiments.

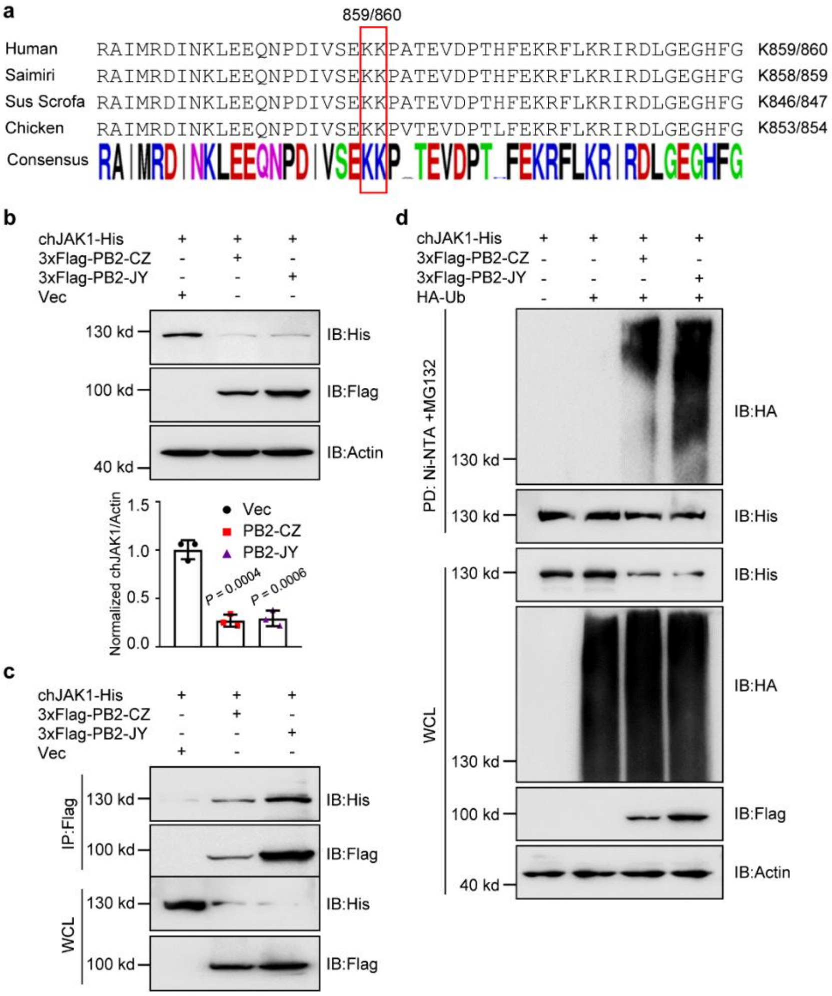

Supplementary Fig. 7 | AIV PB2 protein mediates ubiquitination degradation of chicken JAK1 (chJAK1). (a) Amino acid alignment of JAK1 from the different species. The red box highlights amino acids 859 and 860 correspond to human JAK1. **(b)** Immunoblots of HEK293T cells transfected with PB2 and chJAK1 plasmids (upper). Densitometry analysis of the ratio of JAK1/Actin on immunoblots from three independent experiments (lower). Data are presented as means ± SD and statistical significance was determined by unpaired two-tailed Student's *t*-test. **(c)** Co-ip analysis of the interaction of PB2 with chJAK1 in HEK293T cells. **(d)** Ni-NTA pull-down analysis of the ubiquitination of chJAK1 in HEK293T cells transfected with chJAK1, HA-Ub, and PB2 plasmids. WCL, whole-cell lysates. Data are one representative of three independent experiments.

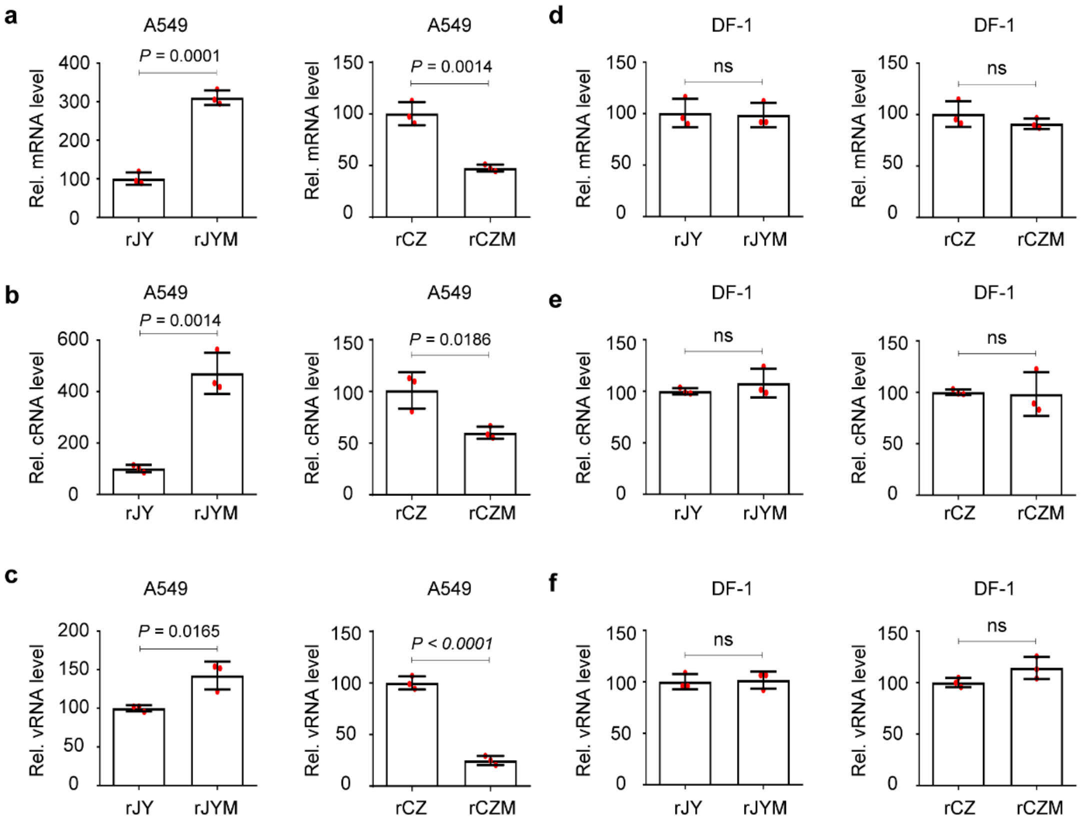

Supplementary Fig. 8 | Quantitative analysis of mRNA, cRNA, and vRNA levels in AIVs-infected cells. (a-c) A549 cells or **(d-f)** DF-1 cells were infected with H5N8 viruses. Levels of NP genes were estimated by quantitative RT-PCR. Data are presented as the mean ± SD and are representative of three independent experiments. ^{ns} $P > 0.05$.

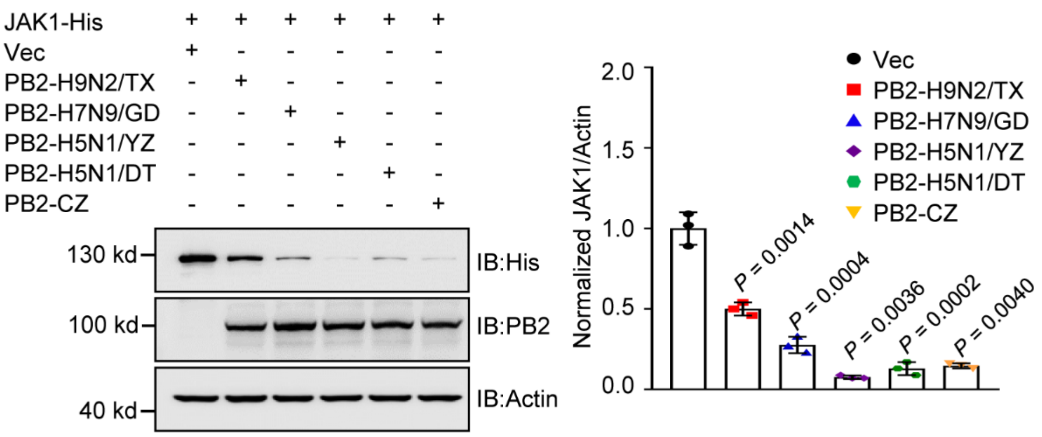

Supplementary Fig. 9 | PB2 protein from different subtypes of AIV promotes degradation of JAK1. Immunoblots of HEK293T cells transfected with different AIV PB2 plasmids (left). The intensities of the bands on the immunoblots from three independent experiments were quantified and normalized with actin (right). Data are presented as means ± SD and statistical significance was determined by unpaired two-tailed Student's *t*-test.

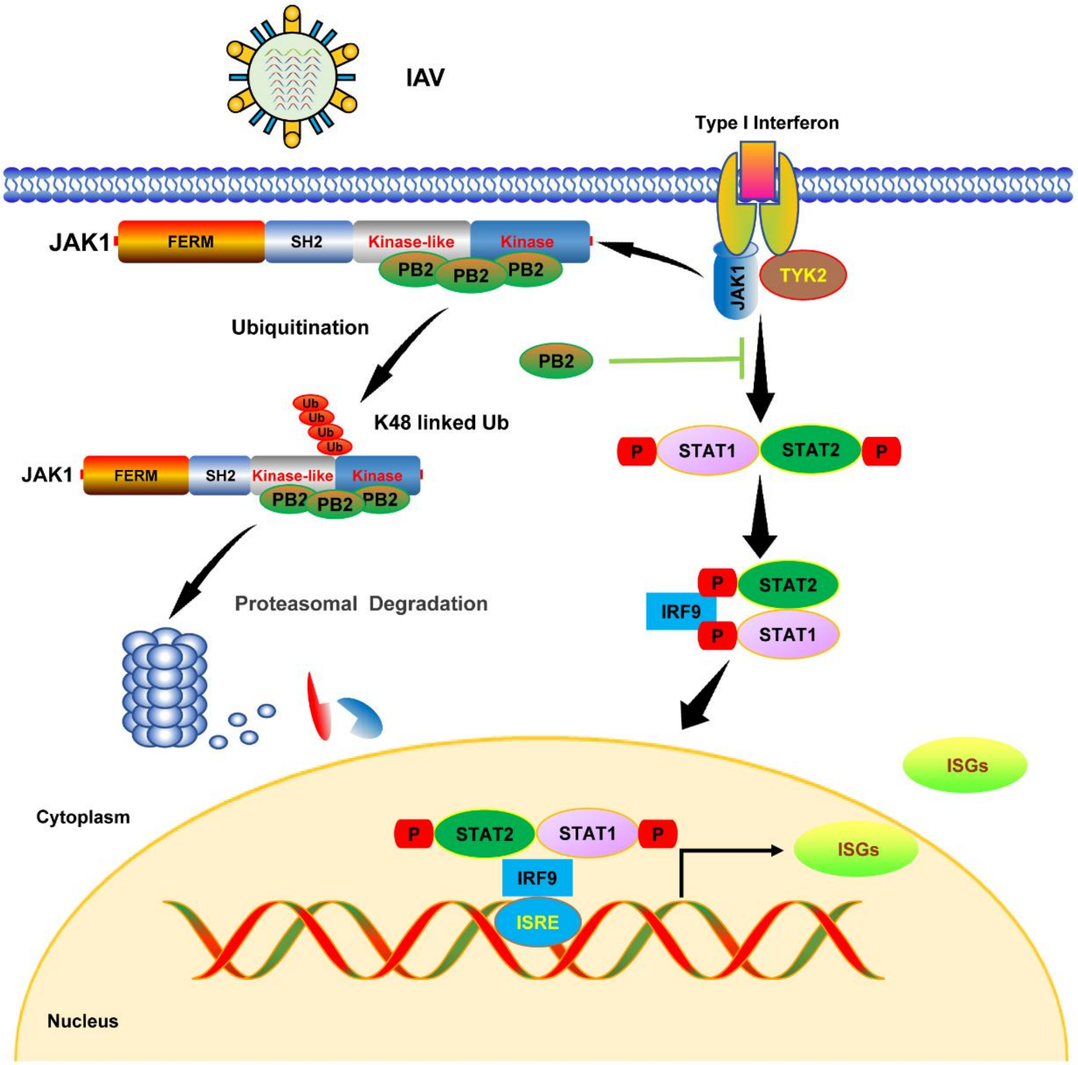

Supplementary Fig. 10 | Schematic representation of IAV PB2 protein antagonizes antiviral immunity. IAV PB2 protein blocks JAK1/STAT signalling by targeting JAK1. IAV PB2 protein mediates K48-linked ubiquitination and proteasomal degradation of JAK1, thereby suppressing JAK1-mediated signal transduction.

2. Supplementary Table 1 | The qPCR primers sequence in this study.

Human Gene		
**Gene**	**Forward (5’-3’)**	**Reverse (5’-3’)**
GAPDH	TCACCACCATGGAGAAGG	GATAAGCAGTTGGTGGTGCA
IFIT1	TCATCAGGTCAAGGATAGTC	CACACTGTATTTGGTGTCTAGG
ISG15	AGGACAGGGTCCCCCTTGCC	CCTCCAGCCCGCTCACTTGC
TAP1	TGTGACAAGGTTCCCACTGCTTAC	GGCTGTGGCCTATGCAGTCA
JAK1	CTTTGCCCTGTATGACGAGAAC	ACCTCATCCGGTAGTGGAGC
**Mouse Gene**		
Actin	GGCTGTATTCCCCTCCATCG	CCAGTTGGTAACAATGCCATGT
ISG15	TGCCTGCAGTTCTGTACCAC	AGTGCTCCAGGACGGTCTTA
IFIT1	CTGAGATGTCACTTCACATGGAA	GTGCATCCCCAATGGGTTCT
**H5N8 virus**		
NP	AGATACTGGGCTATAAGAAC	GCATTGTCTCCGAAGAAATAAG
