# Supplementary material for: The influenza virus PB2 protein evades antiviral innate immunity by inhibiting JAK1/STAT signalling
Source: Nat Commun. 2022 Oct 21;13:6288. doi: 10.1038/s41467-022-33909-2 (PMC9586965; doi:10.1038/s41467-022-33909-2)
Supplement: Supplementary file 3 — Reporting Summary [file 41467_2022_33909_MOESM3_ESM.pdf]

nature portfolio

Corresponding author(s): Daxin Peng

Last updated by author(s): Sep 9, 2022

Reporting Summary

Nature Portfolio wishes to improve the reproducibility of the work that we publish. This form provides structure for consistency and transparency in reporting. For further information on Nature Portfolio policies, see our Editorial Policies and the Editorial Policy Checklist.

Statistics

For all statistical analyses, confirm that the following items are present in the figure legend, table legend, main text, or Methods section.

n/a	Confirmed	
☐	☒	The exact sample size (*n*) for each experimental group/condition, given as a discrete number and unit of measurement
☐	☒	A statement on whether measurements were taken from distinct samples or whether the same sample was measured repeatedly
☐	☒	The statistical test(s) used AND whether they are one- or two-sided<br>*Only common tests should be described solely by name; describe more complex techniques in the Methods section.*
☒	☐	A description of all covariates tested
☒	☐	A description of any assumptions or corrections, such as tests of normality and adjustment for multiple comparisons
☐	☒	A full description of the statistical parameters including central tendency (e.g. means) or other basic estimates (e.g. regression coefficient) AND variation (e.g. standard deviation) or associated estimates of uncertainty (e.g. confidence intervals)
☐	☒	For null hypothesis testing, the test statistic (e.g. *F*, *t*, *r*) with confidence intervals, effect sizes, degrees of freedom and *P* value noted<br>*Give P values as exact values whenever suitable.*
☒	☐	For Bayesian analysis, information on the choice of priors and Markov chain Monte Carlo settings
☒	☐	For hierarchical and complex designs, identification of the appropriate level for tests and full reporting of outcomes
☒	☐	Estimates of effect sizes (e.g. Cohen's *d*, Pearson's *r*), indicating how they were calculated

Our web collection on statistics for biologists contains articles on many of the points above.

Software and code

Policy information about availability of computer code

Data collection	BD Biosciences<br>Leica SP8 confocal microscope
Data analysis	GraphPad Prism V8.0.1<br>LAS X Software V3.7.4<br>FlowJo V10 Software

For manuscripts utilizing custom algorithms or software that are central to the research but not yet described in published literature, software must be made available to editors and reviewers. We strongly encourage code deposition in a community repository (e.g. GitHub). See the Nature Portfolio guidelines for submitting code & software for further information.

Data

Policy information about availability of data

All manuscripts must include a data availability statement. This statement should provide the following information, where applicable:

- Accession codes, unique identifiers, or web links for publicly available datasets
- A description of any restrictions on data availability
- For clinical datasets or third party data, please ensure that the statement adheres to our policy

Source Data are provided with this paper and all relevant data are available from the authors.The data used to generate the image in Fig. 4f are available from PBD

entry 4ehz (http://www.rcsb.org/structure/4ehz).

Human research participants

Policy information about studies involving human research participants and Sex and Gender in Research.

Reporting on sex and gender	N/A
Population characteristics	N/A
Recruitment	N/A
Ethics oversight	N/A

Note that full information on the approval of the study protocol must also be provided in the manuscript.

Field-specific reporting

Please select the one below that is the best fit for your research. If you are not sure, read the appropriate sections before making your selection.

[x] Life sciences [ ] Behavioural & social sciences [ ] Ecological, evolutionary & environmental sciences

For a reference copy of the document with all sections, see nature.com/documents/nr-reporting-summary-flat.pdf

Life sciences study design

All studies must disclose on these points even when the disclosure is negative.

Sample size	Generally, there was no predetermination of sample size for this study. Where required for statistical analyses, sample size included at least three independent biological replicates. The number of required technical replicates and independent biological replicates that were performed was determined, as common in the field, by the requirement for statistical significance. Details regarding sample size of these and all other performed experiments are provided in the legends of figures, the supplementary information and the Methods section.
Data exclusions	No data were excluded from the analyses.
Replication	Animal studies were performed twice and other experiments were conducted at least three times independently. All replication attempts were successful.
Randomization	All samples were randomly assigned to experimental groups.
Blinding	Blinding was not performed in most of the experiments of this study, as most phenotypes observed have obvious differences. The lung histological scores were measured in a blinded manner.

Reporting for specific materials, systems and methods

We require information from authors about some types of materials, experimental systems and methods used in many studies. Here, indicate whether each material, system or method listed is relevant to your study. If you are not sure if a list item applies to your research, read the appropriate section before selecting a response.

Materials & experimental systems

n/a	Involved in the study	
[ ]	[x]	Antibodies
[ ]	[x]	Eukaryotic cell lines
[x]	[ ]	Palaeontology and archaeology
[ ]	[x]	Animals and other organisms
[x]	[ ]	Clinical data
[x]	[ ]	Dual use research of concern

Methods

n/a	Involved in the study	
[x]	[ ]	ChIP-seq
[ ]	[x]	Flow cytometry
[x]	[ ]	MRI-based neuroimaging

Antibodies

Antibodies used	Primary antibody:

Mouse anti-NP mAb (GTX629633, 1:1000) and rabbit anti-PB2 pAb (GTX125926, 1:1000) were purchased from GeneTex. Rabbit anti-STAT1 mAb (14995S, 1:1000), rabbit anti-pSTAT1 mAb (8826S, 1:1000), rabbit anti-STAT2 mAb (72604S, 1:1000), rabbit anti-pSTAT2 mAb (88410S, 1:1000), mouse anti-Ubiquitin (Ub) mAb (3936S, 1:500), rabbit anti-K48-linkage specific polyubiquitin mAb (8081S, 1:500), mouse anti-HA tag mAb (2367S, 1:500), rabbit anti-JAK1 mAb (29261S, 1:1000) and mouse anti-JAK1 mAb (50996S, 1:1000) were purchased from Cell Signaling Technology. Rabbit anti-beta Actin pAb (Abcam, ab8227, 1:1000); mouse anti-beta Actin mAb (Santa Cruz, sc47778, 1:2000), mouse anti-JAK1 mAb (Zen BIO, 200622-8B8, 1:500), rabbit anti-Interferon alpha/beta receptor 1 pAb (Abcam, ab245367, 1:500), mouse anti-His tag mAb (HUABIO, M0812-3, 1:2000), rabbit anti-Ub mAb (HUABIO, ET1609-21, 1:500) and mouse anti-Flag M2 mAb (Sigma-Aldrich, F1804, 1:2000) were purchased from the indicated manufacturers.
Secondary antibody:
Alexa Fluor™ 488 Goat anti-Mouse IgG (H+L) (A11029, 1:400), Alexa Fluor™ 594 Goat anti-Rabbit IgG (H+L) (A11037, 1:400) and Alexa Fluor™ 488 Goat anti-Rabbit IgG (H+L) (A11034, 1:400) were purchased from Invitrogen.

Validation

Validation of the use of NP antibody for Influenza A virus in IHC and WB has been provided by the manufacturer's website. https://www.genetex.cn/Product/Detail/Influenza-A-virus-Nucleoprotein-antibody-GT1236/GTX629633

Validation of the use of PB2 antibody for Influenza A virus in WB has been provided by the manufacturer's website. https://www.genetex.cn/Product/Detail/Influenza-A-virus-PB2-protein-antibody/GTX125926

Validation of the use of STAT1 antibody for human in WB has been provided by the manufacturer's website. https://www.cellsignal.cn/products/primary-antibodies/stat1-d4y6z-rabbit-mab/14995?site-search-type=Products&N=4294956287&Ntt=stat1&fromPage=plp

Validation of the use of STAT2 antibody for human in WB has been provided by the manufacturer's website. https://www.cellsignal.cn/products/primary-antibodies/stat2-d9j7l-rabbit-mab/72604?site-search-type=Products&N=4294956287&Ntt=stat2&fromPage=plp

Validation of the use of pSTAT1 antibody for human in WB has been provided by the manufacturer's website. https://www.cellsignal.cn/products/primary-antibodies/phospho-stat1-ser727-d3b7-rabbit-mab/8826?site-search-type=Products&N=4294956287&Ntt=pstat1&fromPage=plp

Validation of the use of pSTAT2 antibody for human in WB has been provided by the manufacturer's website. https://www.cellsignal.cn/products/primary-antibodies/phospho-stat2-tyr690-d3p2p-rabbit-mab/88410?_=1662526782926&Ntt=PStat2&tahead=true

Validation of the use of Ubiquitin antibody for human in WB has been provided by the manufacturer's website. https://www.cellsignal.cn/products/primary-antibodies/ubiquitin-p4d1-mouse-mab/3936?site-search-type=Products&N=4294956287&Ntt=ub&fromPage=plp

Validation of the use of K48-linkage Specific Polyubiquitin antibody for human in WB has been provided by the manufacturer's website. https://www.cellsignal.cn/products/primary-antibodies/k48-linkage-specific-polyubiquitin-d9d5-rabbit-mab/8081?site-search-type=Products&N=4294956287&Ntt=k48-linkage+specific+polyubiquitin&fromPage=plp

Validation of the use of HA tag antibody in WB has been provided by the manufacturer's website. https://www.cellsignal.cn/products/primary-antibodies/ha-tag-6e2-mouse-mab/2367?site-search-type=Products&N=4294956287&Ntt=ha&fromPage=plp

Validation of the use of JAK1 antibody for human and mouse in WB has been provided by the manufacturer's website. https://www.cellsignal.cn/products/primary-antibodies/jak1-e3a6m-rabbit-mab/29261?site-search-type=Products&N=4294956287&Ntt=jak1&fromPage=plp

Validation of the use of JAK1 antibody for human and mouse in WB has been provided by the manufacturer's website. https://www.cellsignal.cn/products/primary-antibodies/jak1-d1t6w-mouse-mab/50996?site-search-type=Products&N=4294956287&Ntt=jak1&fromPage=plp

Validation of the use of JAK1 antibody for human in WB and IF has been provided by the manufacturer's website. http://www.zen-bio.cn/prod_view.aspx?IsActiveTarget=True&TypeId=150&Id=480963&FId=t3:150:3

Validation of the use of beta Actin antibody for human and mouse in WB has been provided by the manufacturer's website. https://www.abcam.cn/beta-actin-antibody-ab8227.html

Validation of the use of beta Actin antibody for human and mouse in WB has been provided by the manufacturer's website. https://www.scbt.com/zh/p/beta-actin-antibody-c4?requestFrom=search

Validation of the use of Interferon alpha/beta receptor 1 antibody for human in WB has been provided by the manufacturer's website. https://www.abcam.cn/interferon-alphabeta-receptor-1-antibody-ab245367.html

Validation of the use of His tag antibody in WB has been provided by the manufacturer's website. http://www.huabio.cn/product/6X-His-tag-C-terminal-antibody-M0812-3

Validation of the use of Ub antibody for human in WB has been provided by the manufacturer's website. http://www.huabio.cn/product/Ubiquitin-antibody-ET1609-21

Validation of the use of Flag tag antibody in WB has been provided by the manufacturer's website. https://www.sigmaaldrich.cn/CN/zh/product/sigma/f1804

Eukaryotic cell lines

Policy information about cell lines and Sex and Gender in Research

Cell line source(s)	Human embryonic kidneys (HEK) 293T cells, the chicken fibroblast cell line DF-1 cells, MDCK cells and human lung epithelial A549 cells were obtained from American Type Culture Collection (ATCC).
Authentication	All cells lines were verified by ATCC. All cell lines were routinely authenticated in our lab by morphological examination using microscopy and were not authenticated again genetically.
Mycoplasma contamination	All cell lines tested negative for mycoplasma contamination.
Commonly misidentified lines (See ICLAC register)	No commonly misidentified cell lines were used.

Animals and other research organisms

Policy information about studies involving animals; ARRIVE guidelines recommended for reporting animal research, and Sex and Gender in Research

Laboratory animals	4-6 week-old female BALB/c mice.
Wild animals	The study did not involve wild animals.
Reporting on sex	To reduce the data variation, only one sex of BALB/c mice was chosen for challenge study.
Field-collected samples	The study did not involve samples collected from the filed.
Ethics oversight	All animal studies follow the protocols of Jiangsu Province Administrative Committee for Laboratory Animals protocols (approval number: SYXK-SU-2017-0044).

Note that full information on the approval of the study protocol must also be provided in the manuscript.

Flow Cytometry

Plots

Confirm that:

- [x] The axis labels state the marker and fluorochrome used (e.g. CD4-FITC).
- [x] The axis scales are clearly visible. Include numbers along axes only for bottom left plot of group (a 'group' is an analysis of identical markers).
- [x] All plots are contour plots with outliers or pseudocolor plots.
- [x] A numerical value for number of cells or percentage (with statistics) is provided.

Methodology

Sample preparation	HEK293T cells were transfected with PB2 or NS1 plasmid and infected with SeV. The supernatants were inactivated by ultraviolet radiation and collected to treat fresh HEK293T cells for 24 h, followed by infection for 12 h with VSV-GFP. The cells were assessed by flow cytometry.
Instrument	BD Biosciences.
Software	Data was analyzed using FlowJo V10 software.
Cell population abundance	10,000 cells per tube were collected.
Gating strategy	All Cells were gated based on size and granularity using FSC-A vs SSC-A. GFP positive cells were identified by comparing with the control samples.

- [x] Tick this box to confirm that a figure exemplifying the gating strategy is provided in the Supplementary Information.
